# The Changing Narratives of Death, Dying, and HIV in the United Kingdom

**DOI:** 10.1177/1049732320922510

**Published:** 2020-06-06

**Authors:** Jose Catalan, Damien Ridge, Anna Cheshire, Barbara Hedge, Dana Rosenfeld

**Affiliations:** 1 South Kensington and Chelsea Mental Health Centre, London, United Kingdom; 2 University of Westminster, London, United Kingdom

**Keywords:** HIV, death literacy, dying, PLWH, health care, HIV activists, neoliberalism, qualitative interviews

## Abstract

Death and infection were closely linked from the start of the HIV epidemic, until successful treatments became available. The initial impact of mostly young, gay men dying from HIV was powerful in shaping UK responses. Neoliberal discourses developed at the same time, particularly focusing on how citizens (rather than the state) should take responsibility to improve health. Subsequently “successful ageing” became an allied discourse, further marginalising death discussions. Our study reflected on a broad range of meanings around death within the historical UK epidemic, to examine how dying narratives shape contemporary HIV experiences. Fifty-one participants including people living with HIV, professionals, and activists were recruited for semistructured interviews. Assuming a symbolic interactionist framework, analysis highlighted how HIV deaths were initially experienced as not only traumatic but also energizing, leading to creativity. With effective antiretrovirals, dying changed shape (e.g., loss of death literacy), and better integration of palliative care was recommended.

## Introduction

From the start of the HIV epidemic in the United Kingdom in the early 1980s, a strong association between infection and death was established in the public mind (Garfield, 1994; Nuland, 1994; Shilts, 1987). At the height of the epidemic between 1992 and 1995, HIV-related deaths in the United Kingdom were around 1,700 annually, subsequently dropping sharply to around 500 in 1998 with the introduction of successful antiretroviral treatments (Brown et al., 2018). One of the most striking features of the HIV epidemic from the beginning was that it tended to affect young, sexually active, previously healthy men who have sex with men (MSM), as HIV was mainly transmitted via anal intercourse in the United Kingdom. The impact of those early deaths on people living with HIV (PLWH), or at risk of HIV, and on their voluntary and professional carers, was cumulate and powerful, and contributed to shaping the responses to the epidemic. Activists worked to create networks of support and education, and to fight discrimination against PLWH. Residential services for palliative and terminal care were set up, creating innovative models of care centered on dealing with the practical, emotional, and social impact of the many deaths that were expected (Cantacuzino, 1993; Richardson & Bolle, 1992; Spence, 1996). Specialist multidisciplinary units dealt with the acute complications of advanced HIV infection, facing a level of death in younger members of the community that many health workers had never experienced before. MSM (the group most heavily affected by the epidemic), PLWH, and those affected by their predicament (including the wider lesbian and gay communities, and health professionals and researchers) developed holistic approaches to caring for those living with HIV and dying with AIDS. In the United States, the now famous “Denver Principles,” drafted in 1983 by the People With AIDS Self-Empowerment Movement’s advisory committee, identified the right of PLWH to be “active and equal partners in the response to HIV and AIDS,” and included the right to “live—and die—with dignity” (Morolake et al., 2009).

Substantial numbers of critically ill people were of similar ages and backgrounds to those looking after them. PLWH shared their fears and emotions with the care teams, often teaching doctors and nurses about end-of-life experiences. The inevitable awareness of death connected to the virus led in many instances to more open conversations about “living wills” and “advance directives,” and to the planning of funerals as celebrations of lives with memorial services to remember those who died. The concept of “death literacy” (that was introduced to highlight the inevitable familiarity with death that was required during these times; Spence, 1996) referred to the “knowledge and skills that [made] it possible to gain access to, understand, and act upon end-of-life and death care options” (Noonan et al., 2016, p. 31). The large number of deaths, and their prominence among previously healthy young men (Rosenfeld et al., 2012), became part of the public imagination. AIDS quilts, comprising panels commemorating individuals who had died of AIDS and sewn by their loved ones, toured nations. In the United States, the 1,920 panels displayed on the U.S. Capitol’s National Mall in 1987, multiplied by a factor of 4 the following year (Blair & Michel, 2007).

Subsequently, the introduction of effective combination antiretroviral therapies (ARTs) after 1996 (Hammer et al., 1997) gradually reduced the high incidence of death among people with HIV. A sense of hope and the possibility of survival led to a gradual shift in the perception of HIV infection from a death sentence to a manageable condition. Eventually, hope developed that survival was not just a possibility, but even likely. The need for palliative and terminal care was seen as less necessary, and some of the well-established services that had been developed in the earlier phase of the epidemic became increasingly redundant over the ensuing years (Harding, 2018). While providing a sense of hope, chronicity tended to weaken the exceptionalism that many had attached to HIV/AIDS historically (Rai et al., 2018). In more recent years, death has receded into the distance, being a less immediate possibility, although mortality is significantly higher among people with HIV compared with the general population for all causes of death (Croxford et al., 2017). Regardless, there is now a greater perception of “normalization” of HIV-related disease, and a focus on how to live, rather than how to die with HIV, which has paradoxically reduced access to palliative care for people with HIV (Harding, 2018).

There is a long literature on aging with HIV (Rosenfeld et al., 2018), and end-of-life care, including for HIV (Harding, 2018), where the *Qualitative Health Research* journal has played a notable role. For example, this literature has examined narratives of suffering of mothers whose sons have died from AIDS (Gregory & Longman, 1992), the difficulties involved in facing life again after confronting death (Trainor & Ezer, 2000), good versus bad deaths (Pierson et al., 2002), the benefits—and difficulties—of acknowledging death for serious illnesses in hospitals (including for HIV; Anderson et al., 2013); as well as the “disqualified” care relationships and grief of homecare workers whose clients die (Tsui et al., 2019). However, as far as we are aware, our article is the first to specifically and comprehensively investigate the meanings underpinning death itself in relation to the historic HIV crisis in the United Kingdom. In the context of contemporary discourses advocating successful aging with HIV (Emlet et al., 2017), our article aimed holistically to examine diverse narratives about death and dying among people with HIV, and others closely involved with the historical epidemic, to shed light on the symbolic world of HIV and death. In particular, we were interested in how neoliberal ideas might influence discourses around aging and dying. These ideas originated earlier in the 20th century, but took hold of Western governments from the 1980s, about when the HIV epidemic was in its infancy. Neoliberalism encouraged “free markets and small states . . . low taxes and lean administration” (Peck et al., 2018, p. 3). Although the concept is fuzzy, as an ideology, it promotes the notion of “freedom” in the sense of reducing the role of the state (e.g., in health care), while placing the emphasis on individuals to become increasingly responsible for monitoring and improving their own health the state is wound back (Asquith, 2009). Some authors have interpreted neoliberalism as a kind of hypercapitalism (Goldin et al., 2014), encouraging us to ignore the structural inequalities that oppress vulnerable groups, who by definition have less control over their health (e.g., Black African people who turn up late for an HIV diagnosis due to high levels of stigma in their communities have a 10-fold higher risk of death in the year of their diagnosis; Brown et al., 2018).

## Method

### Approach

The study approach was qualitative, using one-to-one interviews to collect personal narratives around living/working with HIV. We chose to elicit narratives, as we considered them to be a rich source of data. Narratives provide people with overarching codes that create and transmit meaning in everyday life, they compel us to listen, as well as to consider the moral dimensions of experience (Greenhalgh & Hurwitz, 1999). Narrative accounts provide a unique window into our worlds, enabling people to account better for experiences of illness in relation to their lives, drawing out more broadly how illness can be understood society wide (Frank, 1995; Hydén, 1997). One-to-one interviews allowed us to secure detailed personal narratives about this sensitive topic. Ethical approval for the study was obtained through the Psychology Department Research Ethics Committee at the University of Westminster. Full written consent from each participant was gained, and a “sensitivity protocol” was in place in case participants became upset in interviews (including steps to provide emotional support to participants; Dickson-Swift et al., 2007).

### Participants and Recruitment

The inclusion criteria meant that participants must have the ability to consent to an interview, and have had many years of personal and/or professional experience with HIV. Exclusion criteria were participants who were acutely ill or suffering a severe mental illness or living outside the United Kingdom. Participants were sampled to include a wide range of experiences, including role (e.g., PLWH, health professional, activist, policy and charity worker), varying length of time in the HIV field/living with HIV, professional or lay, health specialty, and employment sector. Participants were recruited, initially working with Catalan and Hedge’s professional networks, and included expert patients (i.e., in the sense that, “knowledge and understanding is derived from experience and not education”; Wilson, 2001), HIV doctors and nurses, people involved in HIV activism or charity work, or who had served on research advisory groups. From here, we used snowball sampling (i.e., participants help to recruit new participants) to ensure the coverage of emergent issues of relevance to the “organic social networks” we were investigating (Noy, 2008, p. 340), including non-London experiences (29% of sample), harder to reach HIV populations (e.g., those with experiences of hemophilia), and unanticipated death-related issues in the narratives, such as related to chemsex (usually covering the use of mephedrone, γ-hydroxybutyrate [GHB], γ-butyrolactone [GBL], and/or methamphetamine to heighten sexual pleasure and reduce challenging feelings). Participants were invited to take part in an interview by email from one of the authors. Fifty-one participants agreed to participate. They were mainly male (69%) and of Caucasian ethnicity (90%), with an average age of 56 years. The majority of PLWH, activists, professionals, and others we interviewed had been active in the field since the late 1980s/early 1990s. See Table 1 for more details.

**Table 1.** Participant sociodemographic information.

Participant sociodemographic profile	*N* (%)	*N* (%)	*N* (%)
	Male	Female	Total
	35 (69)	16 (31)	51
Ethnicity			
Caucasian	34 (97)	12 (75)	46 (90)
African/Afro-Caribbean	1 (2)	2 (12)	3 (6)
South East Asian/ Indian	0 (0)	2 (12)	2 (4)
HIV status			
Positive	18 (51)	2 (13)	20 (39)
Negative or untested	17 (49)	14 (88)	31 (61)
Role^a^			
Health professionals	14 (40)	10 (63)	24 (47)
Activist	8 (23)	0 (0)	8 (16)
Charity worker	3 (9)	1 (6)	4 (8)
Patient	18 (60)	2 (13)	20 (39)
Other^b^	6 (17)	3 (19)	9 (18)
Mode of transmission^c^			
MSM	13 (35)	0 (0)	13 (26)
Heterosexual	1 (3)	2 (13)	3 (6)
Injecting drug use	2 (6)	0 (0)	2 (4)
Hemophilia	2 (6)	0 (0)	2 (4)

*Note.* HIV = human immunodeficiency virus; MSM = men who have sex with men.

a Participants may have more than one role.

b Journalist/politician/clergy/academic.

c Self-reported as most likely.

### Procedure

Interviews were conducted by Cheshire, Hedge, and Catalan (over a 2 year period), all of which were face-to-face, and carried out at a time and place most convenient to participants, for example, homes, university rooms, or work offices. A semistructured interview approach was used. Participants were encouraged to tell their own story of working/living with HIV from their initial involvement to the present time, and to elaborate on death and dying therein. Interviews were conducted between April 2016 and April 2018, and they were recorded and transcribed verbatim by a professional transcription agency that signed a confidentiality agreement. Interviews lasted on average 81 minutes, with a range of 20 to 196 minutes. Transcripts were all checked for accuracy by Cheshire, and identifying names and places were removed from the data. Transcripts were returned to the participants for checking (corrections and any additional comments).

### Analysis

Our article assumed a pragmatic theoretical orientation, namely, symbolic interactionism, focusing on the meanings generated in everyday (micro) social interactions and environments that create symbolic worlds, and how such meanings subsequently direct actions (Stryker, 2017). Data were analyzed iteratively and inductively (Bowling, 2014), using a broadly thematic approach (Braun & Clarke, 2006). NVivo software was used to explore and ask questions of the data. Initially, Catalan immersed himself in the data on death and dying. In particular, he interrogated the data for different time periods (e.g., early days of HIV, post development of effective therapies, current times) and by participant’s role (e.g., PLWH, health professional, activist) to look for contrasts. Key themes were initially written up by this author, and then discussed and debated by all the authors in meetings and via email. Ridge then worked with the themes to further elaborate them and their links, and develop the main concepts informing the article (e.g., symbolic interactionism, neoliberalism). In the ensuing discussions, emergent categories (e.g., grief, trauma, death literacy) were elaborated at length by the authors, and multiple drafts of this article were subsequently developed, and commented on by all authors, to arrive at a more robust analysis. Preliminary analysis were also presented at the AIDS Impact Conference in Cape Town in 2017 by Catalan, and the Men Who Have Sex with Men, Seven to Seventy Conference, Birmingham, by Ridge, also in 2017. These presentations received valuable feedback that further clarified emerging analysis. We met monthly or bimonthly throughout the analysis, and communicated electronically in between. Catalan, Ridge, and Hedge had worked in the HIV sector since the beginning of the epidemic, and both Catalan and Hedge had worked in the United Kingdom since the 1980s. They developed services and carried out research on the impact of HIV on PLWH and their professional carers in large teaching and clinical HIV centers over decades. As a result, the authors had extensive links with—and knowledge of—many of the key individuals involved in HIV, or who were themselves HIV positive.

## Results

### Death as Traumatic and Energizing

Discussions around death and dying most commonly focused on the pre-ART era. At that time, death was an inescapable reality for PLWH, their communities, and the National Health Service (NHS). Nevertheless, it was apparent that this intense phase of the epidemic was at once traumatic and enlightening (as discussed below). As such, it cast a long shadow in the participants’ narratives, even energizing and animating modern-day narratives, although not always articulated clearly by participants as such. One heterosexual woman living with HIV said:

One World AIDS Day I was standing listening to it all and I thought. Oh my God, they are talking about these dead people, and I thought, how depressing is that? Excuse me, I am alive! I was so angry with this death thing.

The initial trauma was to do with being overwhelmed by the sheer number of deaths surrounding everyone involved in HIV and its care. And, it was not just the number of deaths that was traumatizing, but also the disturbing way in which many people tended to die. Deaths were frequently recalled as especially “awful” and unpleasant, involving considerable pain, vomiting and diarrhea, or breathing difficulties, among other distressing symptoms. Gay men living with HIV noted:

There were 12 of us on the ward, and in one week 8 died . . . in those days we were told to get ready to die, and we were dying, and dying in the most dreadful ways . . . it makes me so angry now, because people forget all those dying then.

I had seen death very close by the time my partner died. At Christmas, when he was allowed home, he wanted a bath in his own bathroom, and I could carry him up four flights of stairs in my arms, because he was about four stone. Is this what is going to happen to me? I had seen other people with monstrous carbuncles of KS^1^ on their face. I managed to hold everything together until after the funeral and then I completely went.

Unsurprisingly then, the ordeals of this era were still in attendance in our interviews, reverberating in the narratives we collected, at times as if lying in wait. The trauma was frequently expressed in emotional upset during interviews, and also conveyed using the metaphor of war, as one male nurse noted:

When I went back to work on the ward I suddenly didn’t know how to take blood pressure . . . I was getting terrible nightmares about the people who had died, and I had to stop for a while. I went back to it of course, but that was the time when I went, oh my God, what the hell has just gone on there? It was like, I know people overuse the term, but it was like a war zone.

Nursing required skills beyond working with the immediate needs of patients, and came to involve dealing with death in all its dimensions, as one female nurse said

We had a 12-bedded unit, and there would be people dying daily, and the nurses also managed the 12-bed mortuary. We would go to work and at the handover you would have a list of alive and dead patients, and for your shift you looked after both, and it meant going down to the mortuary and taking bodies out of the fridge and moving them to the viewing room.

However, for those working in HIV, many of whom came from the same communities being devastated by the virus, grief and feelings such as sadness were put to one side to deal with the unrelenting pressure to support and care for the sick. Clearly, looking after people with HIV in the wards and clinics in this pressurized environment took its toll. Here, nursing staff constantly had to undertake work in the wards for which they had not been specifically trained, so they adapted their approaches as they went along, as one male nurse observed,

I had this guy come in, he was so frightened . . . I later sat on the edge of his bed, and he had his head on my chest because I was trying to comfort him, stroking his hair, you wouldn’t get away with it today, but it was a beautiful moment. Yes, he died, and I was 21, and he was the same age as me.

Similarly, doctors had to confront the reality of high death rates among their patients. Trained to diagnose and cure, instead they were unable to avoid the prospect of deterioration and death, as a male doctor remarked,

You had to deal with the fact that people were going to die, you had to prepare them to die because they knew they would die . . . you didn’t avoid talking about it, you had to address where they wanted to die—at home, or in hospital or in a hospice.

For some doctors, the experiences of death led to a resolve to find ways to stop the epidemic in ways that were considered unconventional at the time, as one male doctor stated,

Yes, it was emotional, it hardened you, and it made many of us more adversarial for the fight, fighting to get drugs tested, evaluated with compassion and ethically . . . We took our strength from our early failures, from the deaths we remembered and the funerals we did or didn’t go to.

Narratives from PLWH we collected were infused with feelings of anger and shock, fear, and dread. There was a sense of decimation of entire friendship and support networks, including those of health professionals, whose friendship networks were frequently drawn from groups affected by HIV. Considerable uncertainty about the best course of health care, and treatment consequences, complicated matters. Treatment decisions were perceived as high stakes affairs, making the difference between life and death, such as in the case of failed treatment approaches that created difficult choices, as one gay man verbalized,

My partner decided to go on the AZT trial, while I decided I didn’t want to take any pills, and within 2 years he started to fade as I got stronger from my cancer. He died in my arms later.

Poignantly, the constant presence and trauma of death meant that living also became especially pressing and valued. Here, the liminal position between death and life was said to open up new possibilities for participants. Being forced to contemplate death led to positive changes, and to an increased focus on what really mattered to people. One male charity worker described death as a factor that resulted in people making changes to their lives in response to their increasing awareness of mortality:

I do think that the specter of death was in a way the magic ingredient . . . we saw this extraordinary transformative process, people were having to face their own mortality and, in the process of doing it, they got good support and people were really figuring out in very new and different ways how to live.

In the midst of the crisis, creative responses took shape, and the medical wards and hospices reportedly became hubs of social support and camaraderie, in contrast to the negative public reactions and stigma portrayed by the media outside the wards. Here, health professionals—like this male doctor—conveyed a sense of the privilege involved in working with people at the end of their lives:

It was an immense privilege to get to know those people towards the end of their lives, and it was a privilege to be part of what was a fantastic model of care . . . I think it gave our lives more meaning . . . and a huge understanding about what was precious about life, and it made it very special.

Funerals and their planning became an important feature of life, and frequently became a creative response to the awful reality of sickness and death. Participants, like this gay man living with HIV, recounted how funerals could become celebrations of life:

The wards were places of great contrast because you had mostly young men who were dying, but at the same time there was an element of laughter . . . we went to a lot of funerals, and they were such positive, colorful things, with show tunes instead of hymns . . . funerals became a celebration of life, not of sadness and loss.

### Lazarus Slowly and Death Continues

At the International AIDS Conference in Vancouver in 1996, the results of research showed the powerful effects of combination therapy (Hammer et al., 1997), beginning a new chapter in the treatment of HIV infection. However, not everybody was convinced that these positive effects would last, and some were concerned that this would be a false dawn. But as people started to take their new medication, many soon began to improve. This became known as the “Lazarus” phenomenon or effect, that is, “a significant improvement in health and functioning as a result of current medication advances” (Thompson, 2003, p. 88). Improvements could be dramatic, almost like coming back from death’s door, although for others, it was more gradual, as it took time to match good medication regimes to individuals, as a gay man—and a heterosexual man—both living with HIV recollected:

I was beginning to think oh God, I‘m probably going to die . . . then the new treatments came, and I know I was one of the first to get them, and the effect, well, people used to refer to it as the Lazarus effect. My immune system started to rebuild, because by this time you had so few T-cells you could give them names.

We were hearing about new treatments, but there had been lots of talk about cures before . . . the first and second drug combinations I went on didn’t work, and it was the third one that very, very slowly started to help. They talked about the Lazarus effect, and it was Lazarus slowly.

The health of PLWH began improving substantially in spite of initial doubts and uncertainties. Some of the services planned at the time to cope with high death rates started to become less relevant. Those workers whose lives had been dominated by HIV and its consequences were able to start looking beyond it, and even think about a day when HIV might cease to be a problem, as a charity worker—and female nurse—both recalled:

The really rapid drop in the number of funerals was such a relief . . . and it also allowed a lot of people who had been volunteering to leave and go off and do other things, to reacquaint themselves with their families or their gardens, or focus on gay adoption, for example.

It was really exciting, perhaps we shall be out of a job, and that will be fantastic.

Specialized HIV hospices and wards, where sick and dying patients had been cared for, began to close or were amalgamated with other health areas around the turn of the century, as a female nurse remembered:

When the charity hospice started, the death rate was high, a lot of people chose to die there, but it finally closed . . . its garden is still there, it was a beautiful place that people loved . . . And one of the funny things was that there were lots of people’s remains in the garden, lots of ashes, and the gardeners sent a memo asking people to stop scattering ashes because they were killing the plants. It was like there was too much blood and bone in the garden!

Although the new treatments were the breakthrough everyone had been hoping for, the good news was often tainted by sadness and regret for those who had not survived long enough to receive them. This was particularly salient for those who were dying at this time. For some, it was too late, and the new treatments failed to help some desperate PLWH, as both a gay man living with HIV—and a charity worker—both evoked vividly:

Post Vancouver it was great for some, but for others it was too late, and that was . . . bittersweet doesn’t begin to describe it, it was almost worse than before, because for those who it was too late for, they read all this good news, but it wasn’t for them.

I was in Vancouver in 1996, and it was really exciting, but my other memory was that at the time a colleague was dying in London, and we were getting daily updates about his health, and he died on the final day of the International Conference.

Thus, in spite of the good news, losses continued to accumulate, and for some, they were especially painful because of the increased hope surrounding them. Regrets at the missed opportunities and poor timing continued over the years, and were particularly difficult because of the reminders of survival all around by then, as one female nurse articulated:

I remember one lady who is still around today, her baby was born with HIV and died when she was 3 months old, and it is still very raw for her, and very difficult for her to hear how well children are doing now.

Despite successful treatments dramatically limiting deaths from HIV, there were still ongoing challenges faced by many at-risk populations, which lead to premature death. As well as physical comorbidities such as cancer and renal disease, ongoing mental health problems and stigma interfered with treatments too. For example, recreational drug use among MSM and other groups can present adherence challenges to life-saving medication regimes (McCall et al., 2015). For MSM in the United Kingdom, chemsex has grown in recent years, leaving a minority of MSM vulnerable to health problems (McCall et al., 2015). The irony of living through the HIV crisis to witness modern chemsex-related deaths was not lost on some participants. For African immigrants however, relatively high levels of ongoing stigma compromise diagnosis and treatment, leading to late diagnosis, which substantially increases the chances of an early death (Brown et al., 2018). Quotes from a male nurse and Black African women highlight these kinds of issues:

One of the guys, he still comes to the clinic now, and he is one of the guys who got through. He was going to die and got through. The poor man . . . every time I see him, I hug him. I won’t let him go . . . It’s changed and there is still the psychological stuff, but of course the big thing now is chemsex. Now we are seeing people die of drugs, which is really hard for me to see. We’ve had four deaths from G[HB] this year.

HIV clinics no longer give you a comprehensive service . . . Some people have never told their GP, simply because if you tell the doctor the whole practice will know, including the rude receptionist and the other characters, the last thing you want them to know is about your business, because maybe they live in the same community . . . you just don’t know.

### Living With HIV, Death Denial, and Other Legacies

Once the concerns about facing relentless death had receded, the focus shifted to “normal” life span with HIV. Current day narratives are predominantly focused, not around death, but toward PLWH learning how to live and age well with the condition. For many, however, who had lived through the pre-ART era, this is a challenging prospect. They had not anticipated having a future, many had been advised to spend their life savings while they could, and they had not worked due to their illness and had no savings or pensions. They suffered life-limiting disabilities and mental health problems. These people had already mentally prepared for the prospect of an early death, as a man living with hemophilia and HIV said:

The harder point for me, and I know for most hemophiliacs, was readjusting to: “No, you are not going to get ill and die at some point . . . you have to stop living like you are going to die soon, because you are not” . . . oh God, I’ve got to work for another 30 years, I was hoping to get ill, retire and die (laughter).

At this time, HIV infection as a manageable and chronic illness, rather than a terminal condition, started to enter the health discourse. However, the concept of “chronic illness” when applied to HIV hides a complex set of situations where individuals and more vulnerable groups still struggle with daily existence, both in terms of their general health and ongoing mental and social difficulties. Despite their struggles being less recognized in prevailing health care discourses of a manageable condition, the exhaustion after many years of ill health, and the aggregate experiences of loss and trauma, made them an ill fit for the reductions in NHS provision that accompanied successful treatments, as a gay man—as well as doctor working with injecting drug users—both highlighted:

I have had HIV for 30 years, I have had a heart bypass, I am just finishing my third fight with cancer. I am used to learning how to survive . . . my life’s work these last 30 years has been surviving.

We have a cohort of people diagnosed for a long time who take their treatment well, but are exhausted by it, by the cumulative bereavements, by losing family and friends, and by feeling that the services around them have changed and probably do not meet their needs.

For many PLWH, the startling reversal in their plight was highlighted by realizing that it was their doctors and nurses, the health professionals who had looked after PLWH for many years, who were now vanishing—retiring or dying. It is a paradox both exhilarating and alarming that professional carers were initially set to outlive PLWH by some considerable time, and now, it is the long-standing PLWH who sometimes experience abandonment by long-standing professionals, as a female nurse said:

Patients talk about their doctors, whom they have been seeing for years, and now the doctors are ageing and retiring, and they never thought they would see their doctors retire . . . we were telling them that they were going to leave us, and in fact, we are leaving them. They do feel a sense of loss and bereavement.

For those diagnosed in the current ART era, death although very much part of the collective consciousness in HIV care was not something that they usually needed to think about in conjunction with their diagnosis, as effective medication was available, as a gay man living with the virus remarked:

I guess death is something I never think about, it is just a case of a) die, or b) take your medication and live. It is a very simple choice.

A number of participants were concerned that effective HIV treatment allowed a “death denying culture” to return, following a period when denial was challenged. There was some unease that with death now being less likely, “the magic ingredient” as we noted previously, of focusing attention on the inevitability of death, and thus better ways of living, had been lost also. The “death literacy” (Spence, 1996) that had been acquired in the early days, which included the possibility of talking about and confronting death more openly than before, was thought to have receded. Health professionals described how contemporary HIV deaths (there continues to be around 500 HIV-related deaths annually in the United Kingdom) could be more difficult to deal with, and seen as failure, due to their preventability and the longer standing relationships with their patients. Two male doctors put it succinctly:

Now it’s . . . death, becoming again something slightly secretive that doesn’t happen or is not meant to happen when it happens.

People with HIV still die, and it is harder for medics now, knowing that the expectation is that HIV is treatable, and that death is not meant to happen.

Despite death being less frequently discussed, the legacy of the inevitability of early deaths, and the specter of trauma surrounding deaths, still permeated the current narratives. For PLWH, the sense of decimation of friendship networks frequently led to social isolation, adding to the difficulties of adjusting to long-term survival in a changed world. PLWH were also left with the memories of the pre-ART era, including the friends they lost, not to mention the loss of their own youth to HIV, as some gay men observed:

I lost my peer group, really, and they haven’t been replaced . . . it would have been nice to have those people with us still.

People post pictures of friends [who have died] on Facebook and I just cannot bear to look . . . I just prefer to have it locked away in a sense.

For PLWH, talking therapies, or discussions with close friends, allowed people to reflect on how they had faced up to their own mortality (e.g., similarities or differences with friends who died pre-ART), as well as new issues about dying, such as those related to recreational drug taking, as one gay male echoed:

We have regular conversations now, and it is about so and so has died, and it’s either heart attack, suicide, but at the core of both of those is drugs.

Others described their lack of tolerance when dealing with the newly diagnosed who had not lived through the “war,” as they well remembered the trauma of the pre-ART years, as a male doctor angrily pronounced:

My willingness to put up with people saying “oh, I can’t take this pill once a day it’s too big” or “I can’t swallow pills,” it’s like, well fuck off and die then . . . you’ve caught an infection, you are going to have to take pills for the rest of your life. Take your pill, and then come back and see me in six months, bugger off.

## Discussion

In line with a symbolic interactionist worldview (Stryker, 2017), we focused on the micro world, and the symbolic meanings that emerged around an unprecedented epidemic in the United Kingdom that linked sex and death. We traced transformations in HIV narratives about death and dying, from the terrible pain of loss and anger of the early days, to the hesitant expectation of change, and finally the acceptance of hope. Within a relatively compressed period of decades, and in response to the changes in the treatment of HIV infection and its prognosis, at once noteworthy and subtle changes in the narratives about death and dying with HIV have taken place, not just in the minds of people with HIV infection, but also among activists, among those formally and informally caring for PLWH, and members of HIV charities. Death, “the magic ingredient,” initially generated trauma but also sparked creative and positive responses that influenced not just PLWH, but also some of their voluntary and professional carers, to really live and better face life’s challenges (Calhoun & Tedeschi, 1990). A strong association between HIV and death was established from the start of the epidemic, shaping both negative public responses to the infection and the fight to provide better, person-centered care for those affected, as well as to find effective ways of treating the condition. As the introduction of effective antiretroviral treatments was followed by their widespread use, with striking results on the prognosis for PLWH, a sense of hope and relief started to emerge, but not without anguish.

Rofes (1998) argued strongly for a post-AIDS perspective, and in colorful language wrote about “stuffing the red ribbon rhetoric” (p. 267), “the Quilt having outlived its purpose” (p. 291), and “leaving aside the funereal feelings” (p. 289). There was some concern in the narratives, however, that the understandable consequence of treatment progress is the return to a death-denying culture. Anxiety (and fear) about death frames human reality, where mortality presents an inescapable existential problem that many prefer to delay dealing with (Wong & Tomer, 2011). In spite of Rofes’ optimism for the generation that lived through the epidemic, earlier losses remained a persistent and emotionally laden theme, summed up by Augustine of Hippo^2^ as “The dead are invisible, they are not absent.” Death is thus never ending, it only shifts shape, for example, into the aging and dying of the first-response HIV care professionals, the newer deaths of gay men from drug use associated with mental health–related problems, or Black African people from delayed diagnoses. We thus must continue to face the less conscious concerns around HIV and death, even among those who have a hopeful, optimistic view of their clinical prognosis. In the case of some gay men in particular, there remains a culture of shame, difficulties with self-acceptance, and seeking refuge in strategies for short-term escape from uncomfortable feelings, via recreational drug or alcohol use or sex. Denial of death in this context may be another way of avoiding difficult feelings linked to the long-term “shadow” cast by HIV infection, not to mention homophobia itself, which linked gayness to death and destruction long before HIV materialized (Downs, 2012; Todd, 2016).

Death also reverberates in the choice to live better and find ways to care for PLWH, something that a number of people touched by the early epidemic discussed. The early years of coping with HIV forced those involved in the struggle to face what was perceived as inevitable death, and to develop ways of confronting and transcending it, a pioneering movement that subsequent progress in the treatment of HIV has weakened. It is understandable that the sense of hope and survival associated with HIV infection today would lead to forgetting poor outcomes and the lessons from the past. But more than this, the dominant discourse of aging with HIV is now consistent with wider neoliberal health and aging discourses, emphasizing normal life expectancy, the potential of “successful ageing with HIV” (assuming adequate ART adherence and good access), being productive, and in health terms, looking toward individualistic approaches, rather than increasing investments in state institutions such as the NHS (Calcagno et al., 2015; Lamb, 2014).

When discussions of death and dying occur in relation to HIV, they tend to focus on returning to positive health, and the role of patient-enterprise therein, rarely taking account of social inequalities that result in not everyone having access to sufficient health care, and social and financial resources that promote opportunities to be healthy (Newman et al., 2007). Discourses such as “successful ageing” (which fit well with neoliberal ideas as the emphasis is placed on individuals, not the state; Rubinstein & de Medeiros, 2014) can promote the idea that “everything is OK” with HIV care, and sideline talk of death, even when the harsh realities of life for many PLWH are there if you look for them. Older PLWH, for instance, experience ongoing anxieties about aging with HIV (Rosenfeld et al., 2014), and there is the potential for negative physical and mental health impact of HIV and ART on aging among the more vulnerable (Vance et al., 2011). There is also increased likelihood of PLWH experiencing comorbidities as they age, whether directly related to HIV or not (Capeau, 2011); not to mention the fact that the “unruly” (HIV) body will inevitably decline and die, regardless of successful aging discourses (Broom, 2016). In contrast to such thinking, earlier HIV discourses assumed some death literacy, and focused on death as inevitable, the sense of “communitas” that those days of the epidemic generated, as well as the ability to recognize the need to face up to death without seeing it as failure (Rai et al., 2018). In addition, the role of the state—able to mobilize and support vast resourcing to address the HIV epidemic—was seen as pivotal in the initial response in the United Kingdom.

Possible reasons for the loss of “death literacy” include the greater potential for a “death denying” culture when mortality became much less common among PLWH, and fewer public ceremonies to commemorate the dead. Interestingly, outside the context of HIV, there has been a growing literature discussing death in medical settings, written by doctors exposed to death on a daily basis. This literature highlights the limits of medical intervention in the context of dealing with progressive conditions and terminal care, and how the complexity of choices and treatments available can distract from the facts at hand (Gawande, 2014; O’Mahony, 2016). As in the earlier days of high rates of mortality in HIV infection, those closely involved in dealing with death in medical settings recognize the desirability of talking about death and naming it, confronting the fears associated with it, and planning ahead (Mannix, 2018).

If we accept that one of the lessons from the early period of the HIV epidemic has been the development of death literacy, it could be argued with Rofes that living with HIV, rather than obsessing about death, is the right approach when the individual is in good health, a period that may well last many years. The problem, however, is about bringing back the earlier death literacy, especially when PLWH develop serious complications and have to consider the reality of an approaching end. The loss of the previous death literacy may make it harder for the individual to make a realistic appraisal now, perhaps falling back on the “fighting talk” and “battling the disease” clichés that can prevent people from discussing end-of-life wishes (Macmillan, 2018). Integrating palliative care into the overall care of people with HIV is recommended to enhance patient-centered care, affirm life and recast dying as normal process (Harding, 2018). Our analysis highlights how contrary to received wisdom, rather than becoming less important overtime, death has merely morphed. Instead of being seen as a marker of failure and decay, we argue it has never been a more essential consideration for living and dying well.

There were some limitations to our study. The insider status of the researchers facilitated the recruitment of participants, and meant participants provided data with trust. However, it is possible that a common worldview was assumed, and some issues—such as conflicting accounts of the 1980s—may be less examined. However, two authors were not part of this UK history (Ridge and Cheshire), and so were able to interrogate the data collection and analysis as “outsiders” at regular meetings, to ensure a more robust article.

## Notes

1. Kaposi Sarcoma, a type of cancer that can cause visible, distinctive purple lesions on the skin.

2. Augustine of Hippo (354 to 430 AD) was a bishop and early Christian theologian whose writings profoundly influenced Western philosophy, and who is now recognized as a saint.

## References

[bibr1-1049732320922510] Anderson W. G. Kools S. Lyndon A. (2013). Dancing around death: Hospitalist–patient communication about serious illness. Qualitative Health Research, 23(1), 3–13. 10.1177/1049732312461728 23034778 PMC3502664

[bibr2-1049732320922510] Asquith N. (2009). Positive ageing, neoliberalism and Australian sociology. Journal of Sociology, 45(3), 255–269. 10.1093/geront/gnu080

[bibr3-1049732320922510] Blair C. Michel N. (2007). The AIDS Memorial Quilt and the contemporary culture of public commemoration. Rhetoric and Public Affairs, 10(4), 595–626. 10.1353/rap.2008.0024

[bibr4-1049732320922510] Bowling A. (2014). Research methods in health: Investigating health and health services. McGraw-Hill Education.

[bibr5-1049732320922510] Braun V. Clarke V. (2006). Using thematic analysis in psychology. Qualitative Research in Psychology, 3(2), 77–101. 10.1191/1478088706qp063oa

[bibr6-1049732320922510] Broom A. (2016). Dying: A social perspective on the end-of-life. Routledge.

[bibr7-1049732320922510] Brown A. Nash S. Connor N. Kirwan P. Ogaz D. Croxford S.. . . Delpech V. (2018). Towards elimination of HIV transmission, AIDS and HIV-related deaths in the UK. HIV Medicine, 19(8), 505–512. 10.1111/hiv.12617 29923668

[bibr8-1049732320922510] Calcagno A. Nozza S. Muss C. Celesia B. Carli F. Piconi S.. . . Ripamonti D. (2015). Ageing with HIV: A multidisciplinary review. Infection, 43(5), 509–522. 10.1007/s15010-015-0795-5 25987480

[bibr9-1049732320922510] Calhoun L. G. Tedeschi R. G. (1990). Positive aspects of critical life problems: Recollections of grief. Omega-Journal of Death and Dying, 20(4), 265–272. https://doi.org/10.2190%2FQDY6-6PQC-KQWV-5U7K

[bibr10-1049732320922510] Cantacuzino M. (1993). Till break of day: Meeting the challenge of HIV and AIDS at London Lighthouse. Heinemann.

[bibr11-1049732320922510] Capeau J. (2011). Premature aging and premature age-related comorbidities in HIV-infected patients: Facts and hypotheses. Clinical Infectious Diseases, 53(11), 1127–1129. 10.1093/cid/cir628 21998279

[bibr12-1049732320922510] Croxford S. Kitching A. Desai S. Kall M. Edelstein M. Skingsley A.. . . Sullivan A. K. (2017). Mortality and causes of death in people diagnosed with HIV in the era of highly active antiretroviral therapy compared with the general population: An analysis of a national observational cohort. The Lancet Public Health, 2(1), e35–e46. 10.1016/S2468-2667(16)30020-2 29249478

[bibr13-1049732320922510] Dickson-Swift V. James E. L. Kippen S. Liamputtong P. (2007). Doing sensitive research: What challenges do qualitative researchers face? Qualitative Research, 7(3), 327–353. https://doi.org/10.1177%2F1468794107078515

[bibr14-1049732320922510] Downs A. (2012). The velvet rage: Overcoming the pain of growing up gay in a straight man’s world. Da Capo Lifelong Books.

[bibr15-1049732320922510] Emlet C. A. Harris L. Furlotte C. Brennan D. J. Pierpaoli C. M. (2017). “I’m happy in my life now, I’m a positive person”: Approaches to successful ageing in older adults living with HIV in Ontario, Canada. Ageing & Society, 37(10), 2128–2151. 10.1017/S0144686X16000878

[bibr16-1049732320922510] Frank A. (1995). The wounded storyteller: Body, illness, and ethics. University of Chicago Press.

[bibr17-1049732320922510] Garfield S. (1994). The end of innocence: Britain in the time of AIDS. Faber and Faber.

[bibr18-1049732320922510] Gawande A. (2014). Being mortal: Medicine and what matters in the end. Profile Books.

[bibr19-1049732320922510] Goldin F. Smith D. Smith M. (2014). Imagine: Living in a socialist USA. Harper Collins.

[bibr20-1049732320922510] Greenhalgh T. Hurwitz B. (1999). Why study narrative? British Medical Journal, 318(7175), 48–50. 10.1136/bmj.318.7175.48 9872892 PMC1114541

[bibr21-1049732320922510] Gregory D. Longman A. (1992). Mothers’ suffering: Sons who died of AIDS. Qualitative Health Research, 2(3), 334–357. 10.1177/104973239200200306

[bibr22-1049732320922510] Hammer S. M. Squires K. E. Hughes M. D. Grimes J. M. Demeter L. M. Currier J. S.. . . Deyton L. R. (1997). A controlled trial of two nucleoside analogues plus indinavir in persons with human immunodeficiency virus infection and CD4 cell counts of 200 per cubic millimeter or less. New England Journal of Medicine, 337(11), 725–733. 10.1056/NEJM199709113371101 9287227

[bibr23-1049732320922510] Harding R. (2018). Palliative care as an essential component of the HIV care continuum. The Lancet HIV, 5(9), e524–e530. 10.1016/S2352-3018(18)30110-3 30025682

[bibr24-1049732320922510] Hydén L. C. (1997). Illness and narrative. Sociology of Health & Illness, 19(1), 48–69. 10.1111/j.1467-9566.1997.tb00015.x

[bibr25-1049732320922510] Lamb S. (2014). Permanent personhood or meaningful decline? Toward a critical anthropology of successful aging. Journal of Aging Studies, 29, 41–52. 10.1016/j.jaging.2013.12.006 24655672

[bibr26-1049732320922510] Macmillan. (2018). https://www.macmillan.org.uk/_images/missed-opportunities-end-of-life-advance-care-planning_tcm9-326204.pdf

[bibr27-1049732320922510] Mannix K. (2018). With the end in mind: Dying, death, and wisdom in an age of denial. Hachette. 10.1080/20502877.2018.1440724 29465300

[bibr28-1049732320922510] McCall H. Adams N. Mason D. Willis J. (2015). What is chemsex and why does it matter? British Medical Journal, 351, Article 5790. 10.1136/bmj.h5790 26537832

[bibr29-1049732320922510] Morolake O. Stephens D. Welbourn A. (2009). Greater involvement of people living with HIV in health care. Journal of the International AIDS Society, 12(1), Article 4. 10.1186/1758-2652-12-4 PMC2664321 19284672

[bibr30-1049732320922510] Newman C. E. Bonar M. Greville H. S. Thompson S. C. Bessarab D. Kippax S. C. (2007). “Everything is okay”: The influence of neoliberal discourse on the reported experiences of Aboriginal people in Western Australia who are HIV-positive. Culture, Health & Sexuality, 9(6), 571–584. 10.1080/13691050701496913 17963097

[bibr31-1049732320922510] Noonan K. Horsfall D. Leonard R. Rosenberg J. (2016). Developing death literacy. Progress in Palliative Care, 24(1), 31–35. 10.1080/09699260.2015.1103498

[bibr32-1049732320922510] Noy C. (2008). Sampling knowledge: The hermeneutics of snowball sampling in qualitative research. International Journal of Social Research Methodology, 11(4), 327–344. 10.1080/13645570701401305

[bibr33-1049732320922510] Nuland S. B. (1994). How we die: Reflections of life’s final chapter. Alfred A. Knopf; Random House.

[bibr34-1049732320922510] O’Mahony S. (2016). The way we die now. Head of Zeus.

[bibr35-1049732320922510] Peck J. Brenner N. Theodore N. (2018). Actually existing neoliberalism. The Sage Handbook of Neoliberalism, 1, 3–15.

[bibr36-1049732320922510] Pierson C. Curtis J. R. Patrick D. (2002). A good death: A qualitative study of patients with advanced AIDS. AIDS Care, 14(5), 587–598. 10.1080/0954012021000005416 12419108

[bibr37-1049732320922510] Rai T. Bruton J. Day S. Ward H. (2018). From activism to secrecy: Contemporary experiences of living with HIV in London in people diagnosed from 1986 to 2014. Health Expectations, 21(6), 1134–1141. 10.1111/hex.12816 30168239 PMC6250870

[bibr38-1049732320922510] Richardson A. Bolle D. (1992). Wise before their time: People with AIDS and HIV talk about their lives. Harper Collins.

[bibr39-1049732320922510] Rofes E. (1998). Dry bones breathe: Gay men creating post-AIDS identities and cultures. Harrington Park Press.

[bibr40-1049732320922510] Rosenfeld D. Bartlam B. Smith R. D. (2012). Out of the closet and into the trenches: Gay male baby boomers, aging, and HIV/AIDS. The Gerontologist, 52(2), 255–264. 10.1093/geront/gnr138 22298746

[bibr41-1049732320922510] Rosenfeld D. Ridge D. Catalan J. (2018). Ageing with HIH. In Westwood S. (Ed.), Ageing, diversity and equality: Social justice perspectives (pp. 259–275). Routledge.

[bibr42-1049732320922510] Rosenfeld D. Ridge D. von Lob G. (2014). Vital scientific puzzle or lived uncertainty? Professional and lived approaches to the uncertainties of ageing with HIV. Health Sociology Review, 23(1), 20–32. 10.5172/hesr.2014.23.1.20

[bibr43-1049732320922510] Rubinstein R. L. de Medeiros K. (2014). “Successful aging,” gerontological theory and neoliberalism: A qualitative critique. The Gerontologist, 55(1), 34–42. 10.1093/geront/gnu080 25161262 PMC4986589

[bibr44-1049732320922510] Shilts R. (1987). And the band played on: Politics, people and the AIDS epidemic. St Martin’s Press.

[bibr45-1049732320922510] Spence C. (1996). On watch: Views from the Lighthouse. Cassell.

[bibr46-1049732320922510] Stryker S. (2017). Symbolic interactionism: Themes and variations. In Rosenberg M. Turner R. (Eds.), Social psychology: Sociological perspectives (pp. 3–29). Routledge.

[bibr47-1049732320922510] Thompson B. (2003). Lazarus phenomena in gay men living with HIV: An exploratory study. Social Work in Health Care, 37(1), 87–114. 10.1300/J010v37n01_05 12921407

[bibr48-1049732320922510] Todd M. (2016). Straight jacket. Random House.

[bibr49-1049732320922510] Trainor A. Ezer H. (2000). Rebuilding life: The experience of living with AIDS after facing imminent death. Qualitative Health Research, 10(5), 646–660. 10.1177/104973200129118705 11066870

[bibr50-1049732320922510] Tsui E. K. Franzosa E. Cribbs K. A. Baron S. (2019). Home care workers’ experiences of client death and disenfranchised grief. Qualitative Health Research, 29(3), 382–392. 10.1177/1049732318800461 30264669

[bibr51-1049732320922510] Vance D. E. McGuinness T. Musgrove K. Orel N. A. Fazeli P. L. (2011). Successful aging and the epidemiology of HIV. Clinical Interventions in Aging, 6, Article 181. 10.2147/CIA.S14726 PMC3147048 21822373

[bibr52-1049732320922510] Wilson P. M. (2001). A policy analysis of the expert patient in the United Kingdom: Self-care as an expression of pastoral power? Health & Social Care in the Community, 9(3), 134–142. 10.1046/j.1365-2524.2001.00289.x 11560729

[bibr53-1049732320922510] Wong P. T. Tomer A. (2011). Beyond terror and denial: The positive psychology of death acceptance. Death Studies, 35(2), 99–106. 10.1080/07481187.2011.535377 24501830

